# Integrative approach for women with fibromyalgia in a Veterans Affairs Medical Center: An observational study

**DOI:** 10.1097/MD.0000000000036285

**Published:** 2023-12-15

**Authors:** Harminder Grewal, Cindy Zhuang, Mahwish Iqbal, Bushra Atta Ur Rehman, Julia Norton, Catherine M. Vernon, Simrita Deol, Steven W. Brose

**Affiliations:** a Veterans Affairs Medical Center, Dayton, OH, USA; b State University of New York (SUNY), Upstate Medical University, Syracuse, NY, USA; c Wright State University Boonshoft School of Medicine, Fairborn, OH, USA; d University of Rochester School of Medicine and Dentistry, Rochester, NY, USA; e Veterans Affairs Medical Center, Syracuse, NY, USA; f Northwestern University, Evanston, IL, USA.

**Keywords:** fibromyalgia, integrative care, pain, women

## Abstract

Fibromyalgia, a complex condition characterized by widespread musculoskeletal pain, presents a significant burden on individuals and healthcare systems. This observational study aims to explore the potential of an outpatient integrative care model for the management of fibromyalgia in women, focusing on personalized goals, patient education, non-pharmaceutical treatments, and lifestyle modifications. The primary objective is to assess patient satisfaction and its correlation with pain, quality of life, depression, and post-traumatic stress disorder (PTSD) symptoms. This pilot study seeks to determine the effectiveness of this model in the alleviation of fibromyalgia-related pain and the improvement of overall well-being. Twenty-five women diagnosed with fibromyalgia participated in a 14-week outpatient treatment program at a Veterans Affairs Medical Center, involving weekly patient-directed, integrative group visits and health coaching. Pre- and post-evaluation questionnaires were administered to assess patient satisfaction, patients’ subjective sense of empowerment in the management of fibromyalgia, and symptom improvement (i.e., pain, quality of life, depression, and PTSD). In addition, the study evaluated the correlation of patient empowerment with symptom improvement. The integrative care model received high patient satisfaction, with a mean score of 8.04 out of 10. Significant pain reduction was observed based on the Numeric Rating Scale (n = 22, *P* < .001). Quality of life showed significant improvement according to the Fibromyalgia Impact Questionnaire (n = 24, *P* = .01). Furthermore, depression symptoms improved significantly, as assessed by Patient Health Questionnaire (n = 24, *P = *.04). However, there was no statistically significant change in PTSD scores (n = 22, *P* = .3). Patient empowerment was strongly correlated with pain reduction (n = 25, r = .78, *P* < .001), quality of life (n = 25, r = .57, *P* < .001), and improvement in depression symptoms (n = 22, r = .50, *P = *.004). Pairwise deletion was used for each outcome. This integrative care model demonstrated promising results in effectively managing fibromyalgia-related pain and enhancing quality of life and depression symptoms in women. This model presents a feasible and potentially effective treatment approach for fibromyalgia. Further research with larger sample sizes and control groups is warranted to validate these findings and encourage broader implementation.

## 1. Introduction

### 1.1. Background/rationale

Fibromyalgia, a complex and challenging condition characterized by widespread musculoskeletal pain,^[1]^ presents a significant burden on individuals and healthcare systems. This paper explores the potential of integrative medicine in managing fibromyalgia, particularly in the context of women Veterans. With a prevalence of approximately 2% among the US population, fibromyalgia disproportionately affects women, including a high-risk population of women Veterans.^[2]^ The Women Wellness Center at the Syracuse Veterans Affairs Medical Center (SVAMC) found that 4.9% of its female patient population had fibromyalgia and the Institute of Federal Health Care identified the 628,000 women Veterans who received care from Veteran Affairs (VA) medical centers nationwide as a high-risk population for musculoskeletal and post-traumatic stress-related complications.^[3]^ Cross-sectional analysis predicted significant association of fibromyalgia with mental health disorders and urged better treatments for women Veterans.^[3]^ Additional studies indicated high co-morbidity of chronic pain, depression, post-traumatic stress disorder (PTSD), and overall poor quality of life (QoL) in this population.^[3]^

Dissatisfaction with standard therapeutic approaches to fibromyalgia at both patient and physician levels calls for better treatment methods.^[4–6]^ Several large retrospective health claims database studies have demonstrated prevalent short- and long-acting opioid use among fibromyalgia patients, ranging from 11.3% to 69%.^[4]^ Chronic opioid use in such patients is associated with poorer outcomes than those receiving non-opioids in observational studies and can lead to dependency and many other adverse effects.^[6,7]^

Our integrative care model incorporated Functional Medicine to facilitate the development of personalized care goals for patients. Central to this approach was patient education and empowerment through the implementation of non-pharmaceutical treatments and lifestyle modifications. While the overall benefits of Functional Medicine have been established in literature,^[8,9]^ its specific application in the treatment of fibromyalgia in women remains an area requiring further investigation.

### 1.2. Objectives

This integrative care model supplements standard therapeutic approaches with anti-inflammatory diet, group educational visits, physical exercises (i.e., aquatic therapy, yoga, qigong, tai chi), and stress-reduction techniques (i.e., biofeedback, meditation). Meta-analyses of randomized controlled trials have confirmed the effectiveness and safety of these approaches in treating fibromyalgia.^[5]^ Recent literature demonstrated that an integrative program focusing on lifestyle changes and psychosocial support yielded satisfactory results in fibromyalgia pain management.^[10]^ Nonetheless, such programs had not been widely available to women Veterans in the United States. This integrative care model emphasized patient education and autonomy, allowing patients to choose their preferred treatment(s). The primary objective was to assess patient satisfaction and its correlation with pain, QoL, depression, and potentially PTSD symptoms. We hypothesized that this model would yield high patient satisfaction, lead to patient empowerment and improvement of symptoms.

## 2. Methods

### 2.1. Study design

**Figure 1.**
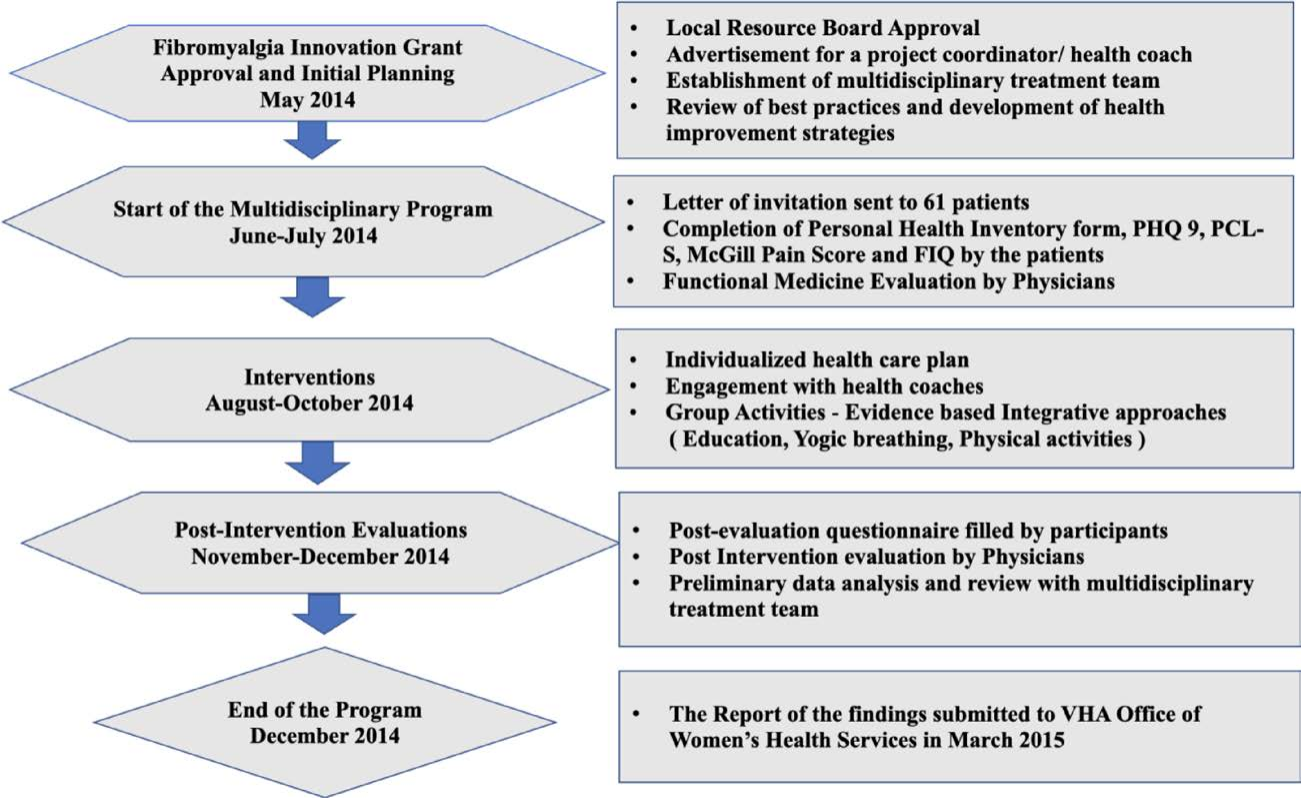
Itinerary of the 14-wk weekly group sessions.

This integrative care model focused on a cohort of women with fibromyalgia. The structured group sessions were designed to enhance health outcomes. The itinerary for these sessions is detailed in Figure 1. Participants were given no financial incentives and were responsible for the copayments for all visits. The retrospective use of patient data for research purposes was approved by the SVAMC Institutional Review Board in accordance with the Declaration of the World Medical Association. Waiver of informed consent for entire program was granted by the SVAMC Institutional Review Board.

### 2.2. Setting

The program was conducted at SVAMC on patients recruited from SVAMC and its associated community-based clinics. The program lasted 8 months. The first 2 months were spent on program development, recruitment, and training of program personnel. Participant recruitment commenced as a letter of invitation to participate in the program was sent.

**Figure 2.**
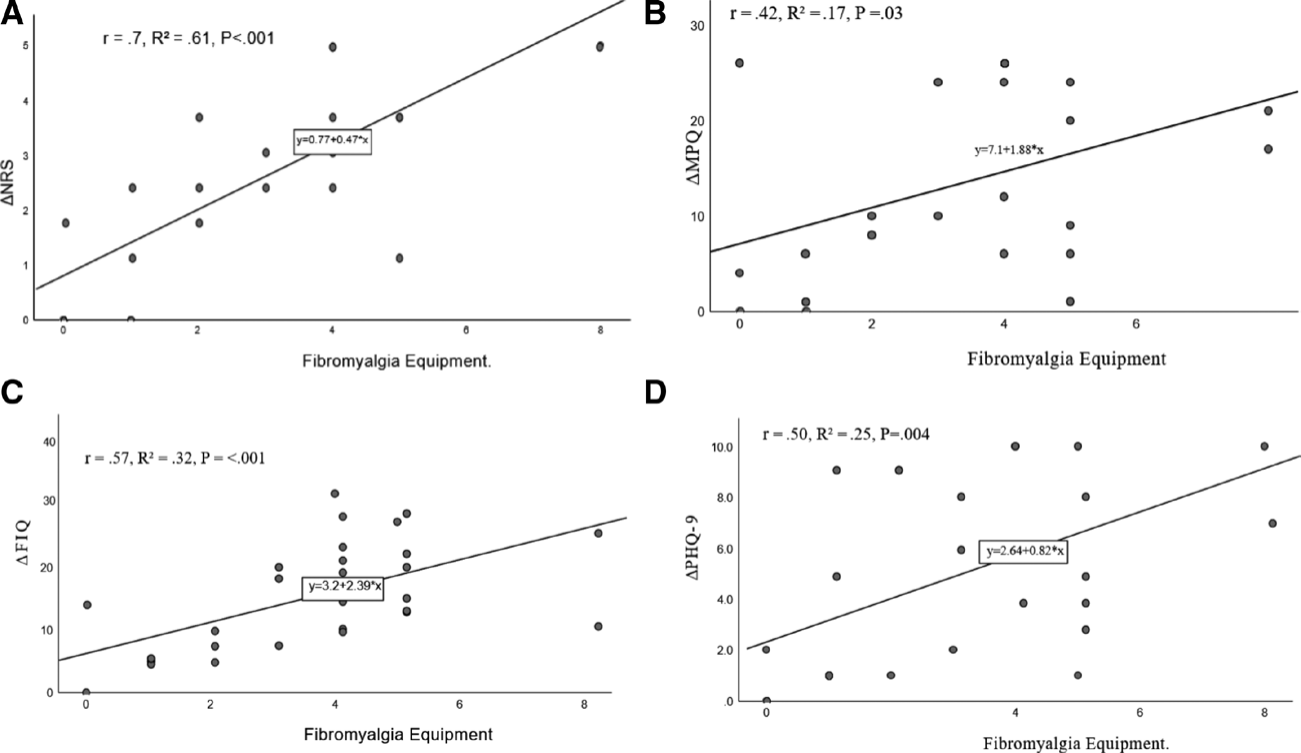
Multidisciplinary Program for Treatment of Fibromyalgia.

Intervention occurred in the following 14 weeks. At baseline and program completion, Pre-Evaluation Questionnaires (Table 1) were administered in conjunction with a physician visit to establish medical history, physical examination, and functional medicine evaluation. Throughout this period, the health coach contacted participants to enhance participation in structured group sessions, which were patient-centered and focused on self-care (Fig. 2).^[11]^ Start and end dates of each phase are detailed in Figure 2.

**Table 1:** Pre-evaluation questionnaire for fibromyalgia treatment program.

Functional medicine evaluation	
General health history	
Fibromyalgia related questions	Fatigue, energy level (i.e., How quickly does it drop? (Suddenly/Slowly)), quality of sleep, aches or pains, mental clarity
Adrenal evaluation	Irritability, shakiness relieved by eating, recurrent infections, severe stress before fatigue started, low blood pressure, feeling dizzy when standing up, frequent craving for sugar, energy improvement in the past with prednisone
Thyroid evaluation	Weight gain/loss, low temperature (under 98F), heavy periods, general feeling of achiness, high cholesterol, unusual sensitivity to cold, dry skin, unusually thin hair, abnormal thyroid function tests or positive antithyroid antibodies
Low estrogen factors	Weight gain/loss, low temperature (under 98F), heavy periods, general feeling of achiness, high cholesterol, unusual sensitivity to cold, dry skin, unusually thin hair, abnormal thyroid function tests or positive antithyroid antibodies
Vasopressor symptom factors	Weight gain/loss, low temperature (under 98F), heavy periods, general feeling of achiness, high cholesterol, unusual sensitivity to cold, dry skin, unusually thin hair, abnormal thyroid function tests or positive antithyroid antibodies
Sleep quality	Problem with sleep, restless legs, snoring, h/o sleep apnea
Sinus/Nasal/Respiratory factors	Chronic nasal congestion, sinus pains, chronic low-grade fevers, congestion
Gastrointestinal factors	Frequent diarrhea, frequent bloating, frequent abdominal pains
Essential fatty acid status	Dry eyes, dry mouth
Neurological factors	Numbness or tingling around the mouth
Behavioral health	Anxiety, panic attacks, depression

Timeline and Key Processes, May 2014 to Dec 2014.

At baseline, to establish personal goal of care, each participant assessed 8 areas of self-care involving physical, mental, social, and spiritual health, using the patient health inventory (PHI) tool developed by the Veterans Health Administration (VHA) Office of Patient-Centered Care and Cultural Transformation.^[12,13]^ Participants also completed the Patient Health Questionnaire-9 (PHQ-9),^[14]^ Post-traumatic Stress Disorder Checklist (PCL-5),^[15]^ McGill Pain Questionnaire 9.5 (MPQ),^[16]^ Fibromyalgia Impact Questionnaire (FIQ),^[17]^ Numeric Rating Scale for Pain (NRS),^[18]^ and Fibromyalgia Equipment—an assessment of how equipped they felt in self-management of their fibromyalgia symptoms.

At the baseline physician visit, full history and physical examination were conducted and questionnaire results reviewed. The history, examination, and questionnaires were standardized using a Functional Medicine model of care to understand the underlying physiological dysfunction leading to disease process. Considering the complexity, chronicity, and multi-system presentation of fibromyalgia, evaluation of adrenal function, thyroid function, female hormones, vasopressor symptoms, sleep quality, sinus/nasal/respiratory factors, gastrointestinal factors, essential fatty acid status, behavioral health, and rheumatological conditions were included. The physicians reviewed all questionnaires and developed a timeline of stressors and their association with symptom development and subsequent diagnosis of fibromyalgia. We utilized a standardized functional medicine matrix tool^[12]^ to categorize the antecedent factors, triggering factors, mediators or perpetuators, changes in physiology and function as defined in Functional Medicine such as assimilation, structural integrity, communication, transport, biotransformation and elimination, energy, defense, and repair.^[12]^ Mental, emotional, spiritual, and personalized lifestyle factors, such as sleep and relaxation, exercise and movement, nutrition and hydration, stress and resilience, and relationships and networks were evaluated and included in the standardized Functional Matrix tool.^[12]^ A clinical note template was developed in the electronic Computerized Patient Record System to document the Functional Medicine patient visit and each matrix was scanned into the respective Computerized Patient Record System patient chart.^[19]^ Additionally, a comprehensive treatment plan template was developed which included both conventional and evidence-based integrative medicine treatment options. Based on the patient preferences, unique history, timeline, physical examination, Functional Medicine evaluation, and PHI/other screening tool responses, the VA collaborative team developed patient-centered treatment plans unique to each patient circumstances, preferences, needs, and disease severity. Patients experienced and learned about the prescribed treatments during the subsequent group visit interventions. These same assessment tools were administered again in the post-evaluation questionnaires to measure subsequent improvements in fibromyalgia related health outcomes.

Options for the treatment plan included conventional treatments and evidence based integrative therapies supported by the VA. The patient potential treatment options included nutrition consults, behavioral health consultations, chiropractic care, physical therapy, physical activities (e.g., yoga, aquatic therapy, qigong), breathing techniques of yoga (e.g., Alternate Nostril Breathing or ANB), and some select supplements available from the VA Pharmacy (e.g., vitamin D, omega-3 fatty acids, and magnesium) based on patient clinical evaluation.^[20]^ The team developed the final plan with the patient using shared decision-making and it included various self-care options. The treatment plan was finalized with the participant using shared decision-making. The health coach helped the patient develop personalized goals of care through determining preferences on the PHI.

Intervention was delivered over 3 months and consisted of: Treatments based on personalized plan, 14 weekly group sessions, and weekly health coaching sessions. Each group session was 2.5-hour long and contained 1 hour of didactic education on topics including fibromyalgia pathophysiology, coping strategies, and anti-inflammatory diet, 30 minutes of ANB exercises,^[21,22]^ and 1 hour of physical activity of each participant choice. Their options included aquatic therapy, taichi, qigong, and yoga. The health coach followed up with participants weekly to assess motivation and enhance treatment adherence. Participants were encouraged to practice the skills learned in group activities at home and were provided with Mayo Clinic Wellness Solutions for Fibromyalgia DVDs.^[23]^

The patients were reevaluated after the end of the group visits and were instructed to complete the Post-Evaluation Questionnaires (Table 2) before this final evaluative visit. They had a comprehensive follow-up physician evaluation to assess the impact of the treatment plan.

**Table 2:** Post-evaluation questionnaire for fibromyalgia treatment program.

• What sessions did you attend for group? Have you been able to incorporate the practices at home?*
• If you did not attend the group, have you been able to incorporate any services for treatment of fibromyalgia at the VA or suggestions for therapies at home?†
• On a scale of 1–10, rate what you have specifically found helpful in the group. (1–10)
• Has your level of pain improved as a result of the practices learned during the session and at home? (1–10)
• Do you feel better equipped to handle fibromyalgia and instill positive change in your life? (1–10)
• Would a support group be something of interest to you for fibromyalgia through the Women Wellness Center and the Pain Clinic? (1–10)
• Have your feelings been validated regarding your fibromyalgia? (1–10)
• How helpful was the initial integrative medicine evaluation and discussion with the integrative doctor? Did it help you get a greater insight into your medical condition and the factors likely contributing to it? (1–10)
• Did you find the self-assessment personal health inventory helpful in understanding yourself and condition? (1–10)
• Did you find the personalized treatment plan useful for you to have? (1–10)
• Did you find the health coaching telephone calls helpful? (1–10)
• Did the health coach motivate you to continue with your health goals and motivate you to address any health opportunities you had planned or considered? (1–10)
• Is having group sessions something we should consider doing in the future for other medical issues such as high blood pressure or depression etc as well? (Yes/No)
• Would you like to have more appointments with integrative medicine physicians for more individualized treatment plan of care? (Yes/No)
• Do you think that the education that you received in the group or with health coaching will motivate you to make and sustain some lifestyle changes? (Yes/No)
• Additional comments

* Options include Pathophysiology of Fibromyalgia, Anti-inflammatory diet, Pain management, Pharmacology/CAM supplements, Guided imagery, Qi gong, Tai chi, Aquatic therapy, HeartMath biofeedback/mindfulness/reduce stress, Acupuncture, Massage therapy/Reiki, Chiropractic care, Aromatherapy/diet, Alpha stimulation, Yoga/meditation, Sleep study.

† Options include Pain clinic/medication, Alpha stimulation, Reiki, Nutrition, Behavioral health, Aquatic therapy, Physical therapy, Supplements, HeartMath biofeedback, Walking/light exercise, Chiropractic care, Yoga/Tai chi/Qi gong, Meditation/guided imagery, Anti-inflammatory diet, Aromatherapy, Massage therapy, Strength exercises, Acupuncture, Sleep study.

### 2.3. Participants

A letter of invitation was mailed to women diagnosed with fibromyalgia and treated at SVAMC or its associated community-based clinics. No other exclusion criteria were applied. Table 3 shows baseline characteristics of the participants. Participants were followed by the health coach weekly.

**Table 3:** Baseline characteristics of the study participants (N = 30).*

Characteristics	Mean (SD)
Mean age, yr	54.3 (9.4)
Race/ethnicity, n (%) • Non-Hispanic white • Non-Hispanic black • Unspecified	23 (76.7) 4 (13.3) 3 (10)
Weight, kg ± SD	89.3 (19.9)
Duration of Fibromyalgia related pain, yr	6.8 (6.6)
Co-morbidities, n (%)† • Musculoskeletal • Psychiatric • Neurological • Cardiovascular • Gastrointestinal	21 (70) 25 (83.3) 10 (33.3) 4 (13.3) 6 (20)

Musculoskeletal: Chronic low back pain (13.3%), osteopenia (6.7%), rheumatoid arthritis (6.7%), spondylosis (13.3%), sciatica (3.3%), systemic lupus erythematosis (3.3%), osteoarthritis (10.0%), sacroiliitis (6.7%), cervicalgia (10.0%), and spinal stenosis (6.7%).

Psychiatric: Depression (36.7%), anxiety (23.3%), PTSD (33.3%), bipolar affective disorder (6.7%), borderline personality disorder (6.7%), and insomnia (6.7%).

Neurological: Migraine (16.7%), carpel tunnel syndrome (6.7%), neuropathy (10.0%), postherpetic neuralgia (3.3%), and trigeminal neuralgia (3.3%).

Cardiovascular: Hypertension (16.7%), obesity (46.7%).

Gastrointestinal: Inflammatory bowel disease (3.3%), irritable bowel syndrome (13.3%), gastroesophageal reflux disease (10.0%).

* For continuous variables, the first number is the mean and parenthetical number is standard deviation. For categorical variables, the first number is the observed count and parenthetical number is weighted percentage.

† Shown are the co-morbidities reported by the study participants.

### 2.4. Variables

Primary outcomes of this program included: Patient satisfaction, defined as patient perceived effectiveness of the program; Self-reported pain levels, measured by MPQ and NRS pain; and QoL, measured by FIQ. Secondary outcomes included: Depression and PTSD severities, measured by PHQ-9 and PCL-5, respectively; Patient-rated most beneficial interventions, defined as the 3 interventions with the highest post-evaluation patient ratings; and Patient satisfaction, evaluated qualitatively through post-evaluation patient comments.

Effects of the intervention might have been confounded by the high rates of musculoskeletal and psychiatric co-morbidities (Table 3) and various degrees of disability among the participants. It was difficult to distinguish if the effects were directed on the pathophysiology of fibromyalgia or indirectly mediated via the effects on other co-morbidities. The multi-systemic nature of fibromyalgia poses challenges to isolating the variables from these confounders.

### 2.5. Data sources/measurement

A primary outcome of this pilot program was patient satisfaction. In the post-evaluation questionnaire, participants were asked to rate the effectiveness of the Functional Medicine evaluation, PHI, treatment plan, and health coaching on a scale of 1 to 10. They were asked to rate their level of motivation to take care of their symptoms after attending health coaching sessions. We asked them whether they felt their symptoms and distress related to fibromyalgia were acknowledged and validated.

Other primary outcomes included the reduction in self-reported pain levels, as measured by 2 well-validated standardized questionnaires (i.e., MPQ and NRS pain), and QoL, as measured by FIQ—a standardized tool to assess the current health status of patients with fibromyalgia. These questionnaires were administered both at baseline and at program completion to compare the difference before and after implementing the interventions.

Secondary outcome measures included depression and PTSD severities, as 83.3% of the participants had psychiatric co-morbidities. Among these participants, depression and PTSD symptoms were evaluated at baseline and at program completion, using the PHQ-9 and PCL-5 questionnaire, respectively.^[18,20,24,25]^ At program completion, patients were asked to rate the degree to which they felt well-equipped in dealing with fibromyalgia as a measure of patient empowerment. We also assessed the impact of patient empowerment on pain levels as measured by MPQ and NRS, fibromyalgia symptoms as measured by FIQ, and depression as measured by PHQ-9.

Interventions perceived to be most beneficial by patients were identified in the post-evaluation questionnaire. In the weekly sessions, the patients were introduced to 17 potential interventions to choose from, including anti-inflammatory diet, pharmacotherapy, essential oils and aroma therapy, biofeedback, relaxation techniques, ANB exercises, mindfulness-based stress reduction, Alpha Stim, chiropractic care, physical therapy, reiki, massage, acupuncture, aquatic therapy, music therapy, and gentle exercises, such as yoga, tai chi, and qigong. Of these, patients had immersive experience in mindfulness-based stress reduction, qigong, tai chi, yoga/meditation, and aquatic therapy.

In addition to the quantitative measures mentioned above, patient satisfaction was evaluated qualitatively using patient comments. Specifically, the post-evaluation questionnaire included an optional open-ended question, “Please write in your own words about your experience,” which allowed the patients to provide any additional comments they might have.

### 2.6. Bias

To avoid Hawthorne effects, we opted to mail the post-evaluation questionnaires to participants for completion at home. However, this approach introduced certain challenges, including potential barriers to questionnaire completion, and resulting in significant missing data. It is important to acknowledge that this missing data could introduce selection bias, as participants with specific characteristics may be more likely to complete the questionnaires.

All questionnaires used were well-validated standardized measurements, thereby minimizing the potential for questionnaire bias. However, considering the extensive use of questionnaires in evaluating outcome measures, we included an optional open-ended question in the post-evaluation phase. This addition aimed to address any unforeseen communication barriers between the investigators and participants, thus mitigating the potential for questionnaire bias. Furthermore, the use of a pre- and post-evaluation design served as an additional safeguard against possible bias.

### 2.7. Study size

Study size was determined by the number of confirmed eligible participants.

### 2.8. Quantitative variables

For patient satisfaction, mean (+/− standard deviation), median, and mode scores of participant-rated effectiveness of Functional Medicine evaluation, PHI, Treatment plan, and Health coaching.

For pain, QoL, depression, and PTSD, mean scores of the corresponding questionnaire (i.e., NRS, MPQ, FIQ, PHQ-9, PCL-5, respectively) were calculated for each participant at baseline and program completion. A linear regression analysis was completed to evaluate the impact of patient empowerment on changes in NRS, MPQ, FIQ, and PHQ-9 scores.

For most beneficial interventions, patients were asked to rate the effectiveness of these interventions for improving their fibromyalgia symptoms on a scale of 1 to 10 in the post-evaluation questionnaire.

### 2.9. Statistical methods

Data were expressed either as frequency (number of subjects/percentage) for categorical variables or as mean (standard deviation) for continuous variables. Patients with missing data were excluded from the analysis of the corresponding variables (i.e., pairwise deletion). This innovation program effects were tested by comparing the difference in mean scores based on pretest and post-test design. All variables passed the normality tests to conduct analysis. Paired sample t-tests were utilized to compare metrics of pain, QoL, depression, PTSD symptoms, and weighted by subtracting post- from pre-intervention evaluations to assess the change during the interventions. The correlation between patient perception of empowerment through multidisciplinary interventions and change in primary outcome measures was assessed using Pearson correlation. *P* < .05 was statistically significant and all tests were 2-tailed. Data management and statistical analysis were performed using the SPSS Statistics Version 26 (IBM Corp., Armonk, NY). Participants who dropped out after completing pre-evaluation questionnaires but did not participate in any group activities were removed from the analysis. Participants who completed all group visits but only some post-evaluation questionnaires were included in the analysis of variables where both pre- and post-evaluation were complete. Numerical Rating Scale, FIQ, Patient Health Questionnaire, and Fibromyalgia Equipment each has 22, 24, 24, and 25 complete cases, respectively. Pairwise deletion was used for each variable.

## 3. Results

### 3.1. Participants

A letter of invitation was mailed to 61 SVAMC female patients diagnosed with fibromyalgia. Thirty-one accepted the invitation. Thirty underwent comprehensive pre-evaluation and development of individualized treatment plans. Six participants dropped out after completing the pre-evaluation questionnaires and did not participate in group sessions, health coaching, or post-evaluation. One of these 6 participants could not participate in group visits due to pregnancy, one due to hearing loss, and 4 felt that their work schedules could not accommodate the group appointments. Twenty-five participants completed the program. Twenty-four completed the post-evaluation. Those who dropped out had similar demographic and co-morbidity profiles as the remaining group.

### 3.2. Descriptive data

**Figure 3.**
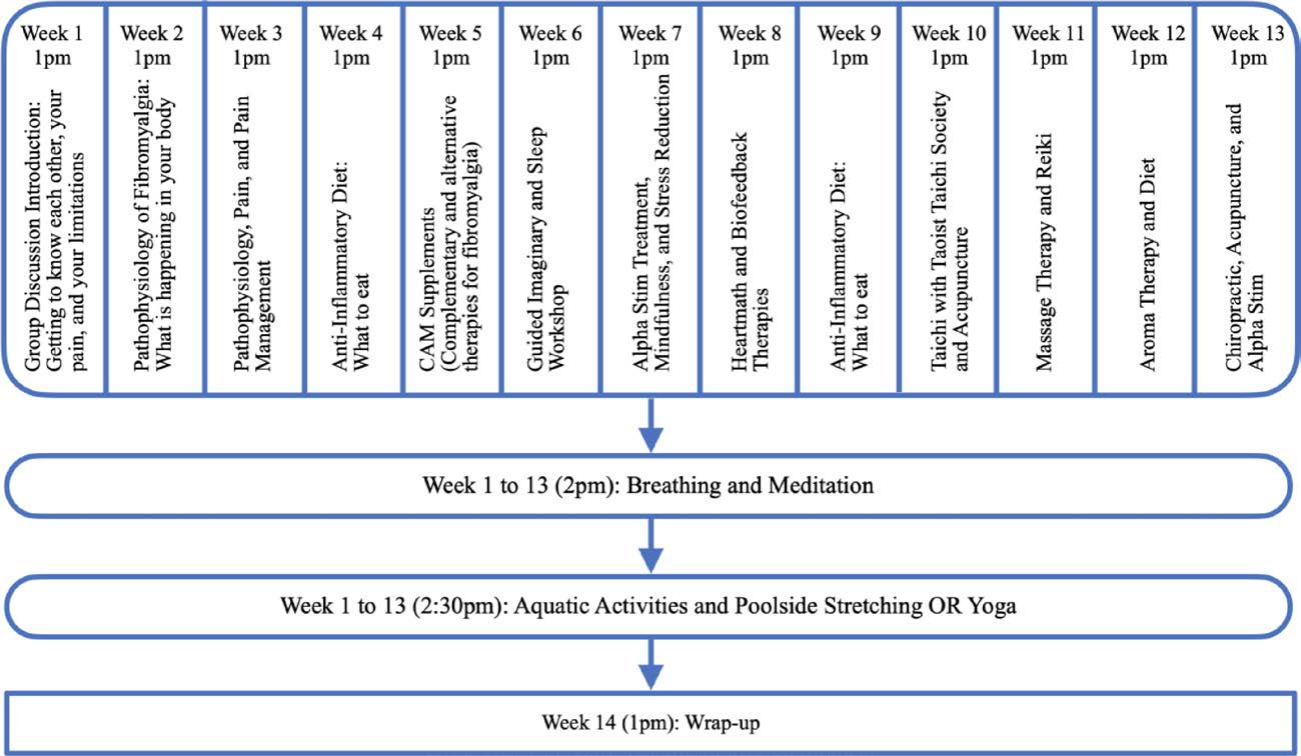
Mean changes in pre- and post-questionnaires pain predicted through Numeric Rating Scale (NRS, range 0–10) and McGill Pain Questionnaire (McGill, range 0–78), in quality of life through Fibromyalgia Impact Questionnaire (FIQ, range 0–100), in depression through Patient Health Questionnaire (PHQ-9, range 0–27), and post-traumatic stress disorder (PTSD) through PTSD Checklist-Specific (PCL-5, range 17–85) and patient perception of empowerment (Fibromyalgia Equipment, FE, range 0–10).

Most participants were Caucasian (76.7%) with a mean age of 54 (range 34–71) years and a mean body weight of 89.3 kg. On average, participants had symptoms of fibromyalgia for 6.8 years, among which, 70% had other musculoskeletal co-morbidities and 83.3% had psychiatric co-morbidities (Table 3). All participants completed the Pre-Evaluation Questionnaires. Figure 3 shows the number of participants who completed both Pre- and Post-Evaluation Questionnaires in each variable of interest. Six participants dropped out after completing the pre-evaluation questionnaires. Among these 6, the mean age was 53.8 +/− 12.8 years, and 83.3% were non-Hispanic White while 16.7% were African American. 66.7% of these 6 participants had psychiatric co-morbidities, and 66.7% of them had musculoskeletal co-morbidities. They had similar demographic and co-morbidity profiles as the remaining group. The remaining 25 participants stayed in the program for 14 weeks.

### 3.3. Outcome data

All outcome measures were assessed at baseline and at program completion.

### 3.4. Main results

Prior to data analysis, we sought consultation from external experts in statistical analysis, methodology, and study design. Data analysis revealed that most of the participants found various aspects of the program highly beneficial, with many reporting positive experiences across all areas. Out of the participants who returned the following pre- and post-evaluation questionnaires, the mean, median, and mode scores for satisfaction were calculated as follows: “Initial Integrative Medicine Evaluation and Discussion” (n = 24): 8.04 +/− 1.67, 8.5, 10; “Self-Assessment Personal Health Inventory” (n = 24): 7.06 +/− 2.86, 8, 10; “Treatment Plan” (n = 19): 8.47 +/− 2.27, 10, 10; “Health Coaching” (n = 17): 8.38 +/− 2.43, 9.5, 10; How motivated the patient felt after health coaching (n = 20): 8.33 +/− 2.50, 9.25, 10; Validation of fibromyalgia related symptoms (n = 24): 8.75 +/− 1.67, 9, 10. Table 4 provides detailed analysis on these scores.

**Table 4:** Patient Satisfaction with the program.

	Initial Eval.	PHI	Treat. Plan	Health Coach	Motivation	Validation
Sample size	24	24	19	17	20	24
Mean score (1–10)	8.04	7.06	8.47	8.38	8.33	8.75
Standard deviation	2.27	2.86	2.27	2.43	2.50	1.67
Median score (1–10)	8.5	8	10	9.5	9.25	9
Mode score (1–10)	10	10	10	10	10	10

Initial Eval. = “Initial Integrative Medicine Evaluation and Discussion”; PHI= “Self-Assessment Personal Health Inventory”; Treat. Plan= “Treatment Plan” (Treat. Plan), Health Coach=“Health Coaching,” Motivation=“How motivated they felt after health coaching”; Validation=“How much their fibromyalgia related symptoms were validated.” The mean, median, and mode scores were measured from low to high (1–10).

The program resulted in significant pain reduction for participants. Based on the NRS, 22 participants reported decreased pain at program completion [95% CI (1.2, 2.7), *P* < .001]. MPQ showed similar results, with 14 participants reporting decreased pain at program completion [95% CI (2.5, 16.4), *P* = .01]. Participants found MPQ confusing and difficult to answer, hence the low completion rate. Overall, the average decrease in pain score was 25.8% and 22.2% based on NRS and MPQ, respectively (Table 5).

**Table 5:** Mean (SD) change in outcome measures from baseline.

Variable	Before Intervention†	After Intervention†	Within-Group Difference, mean	95% CI‡	*P* value‡
Pain					
NRS (n = 22)	7.7 (2.1)	5.7 (1.8)	2.0	(1.2–2.7)	<.001
MPQ (n = 14)	42.7 (6.7)	33.2 (9.7)	9.5	(2.5–16.4)	.01
Quality of Life					
FIQ (n = 24)	68.8 (13.9)	61.8 (17.2)	7.0	(1.5–12.4)	.01
Depression					
PHQ-9 (n = 24)	13.4 (4.7)	11.3 (6.0)	2.1	(0.03–4.1)	.04
PTSD					
PCL-5 (n = 24)	43.1 (15.9)	40.3 (16.4)	2.7	(2.8–8.3)	.3
Patient empowerment					
FE (n = 25)	5.09 (2.44)	8.52 (0.94)	-3.7	(-5.0, -2.4)	.017

† Each participant was assessed for pain (Numerical Rating Scale, NRS, and McGill Pain Questionnaire, MPQ), quality of life (Fibromyalgia Impact Questionnaire, FIQ), depression (Patient Health Questionnaire-9, PHQ-9), and post-traumatic stress disorder (PTSD-stressor Checklist, PCL-5) with standardized questionnaires at baseline and at program completion.

‡ *p* values and 95% CI were calculated with paired sample *t* test.

Similarly, the participants showed significant improvement in QoL at program completion. This was demonstrated by decreased FIQ score from baseline to post-intervention [95% CI (1.5, 12.4), *P *= .01] for the 24 study participants who completed all pre- and post-evaluation questionnaires (Table 5).

Improvement in depression and PTSD symptoms was observed at program completion. The 24 participants who completed all questionnaires scored lower on PHQ-9 than their baseline, with a mean decrease of 15.6% [95% CI (0.03, 4.1), *P* < .05]. Among the 23 participants who had documented PTSD, PCL-5 score decreased by 6.36% (Table 5); however, this decrease was not statistically significant [95% CI (2.8, 8.3), *P* = .3].

**Figure 4.**
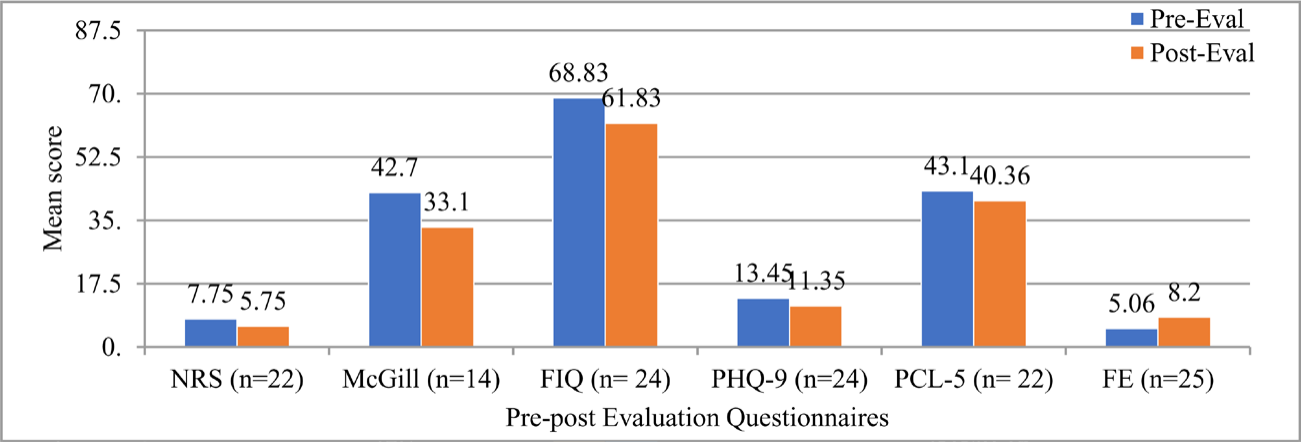
(A) Correlation between patient perception of empowerment (Fibromyalgia Equipment) and changes in primary outcomes. (A) Numeric Rating Scale (NRS) and (B) McGill Pain Questionnaire (MPQ). Middle diagonal line represents the “best fit” linear regression of data with linear regression equation labeled in graphs labeled as A and B. Pearson correlation coefficient (*r*), the coefficient of determination (*R*^2^) and *P* values is shown in the top left corner. Pearson correlation analysis shows a significant positive correlation of patient perception of empowerment with multidisciplinary treatment approaches versus changes in pain scores predicted through (A) NRS (n = 25, *r* = .78, *P* < .001) and (B) MPQ (n = 22, *r* = .42, *P* = .03). MPQ = McGill Pain Questionnaire 9.5. (B) Correlation between patient perception of empowerment (Fibromyalgia Equipment) and changes in primary and secondary outcomes. (C) Fibromyalgia Impact Questionnaire (FIQ) and (D) Patient Health Questionnaire (PHQ-9). Pearson correlation analysis shows a significant positive correlation of patient perception of empowerment with multidisciplinary treatment approaches vs changes in pain scores predicted through (C) FIQ (n = 25, *r* = .57, *P* < .001) and (D) PHQ-9 (n = 22, *r* = .50, *P* = .004).

Scatter plots (Fig. 4A) utilizing a Pearson correlation analysis show a significant positive correlation (R^2^ = 0.61, *R* = 0.78, *P < *.001) between patient perception of empowerment before and after interventions (Fibromyalgia Equipment) and a decrease in NRS pain scores, suggesting strong association between patient empowerment and decrease in pain. Similarly, patient empowerment was significantly correlated (R^2^ = 0.17, *R* = 0.42, *P *= .03) with decrease in MPQ pain scores. A moderate correlation was found between patient empowerment and improvement in QoL (R^2^ = 0.32, *R* = 0.57, *P < *.001), as between patient empowerment and improvement in depression (R^2^ = 0.25, *R* = 0.50, *P *= .04) (Fig. 4B).

Data analysis revealed strong patient preferences for some interventions over others. Based on questionnaire results at program completion, the top 3 treatment interventions that patient noted to be most beneficial were Aquatic Therapy (24%), Yoga/Stretch/Physical Therapy (14%), and Anti-inflammatory Diet/Supplements (12%).

## 4. Discussion

### 4.1. Key results

This pilot program, involving 25 participants, successfully used an integrative medicine model for treatment of fibromyalgia, resulting in notable improvement in symptoms, patient satisfaction, and empowerment.

### 4.2. Limitations

This program used a convenience sample and was designed as a clinical collaborative initiative. Given the high prevalence of co-morbidities in fibromyalgia and the inclusion of patients experiencing non-fibromyalgia-related pain events (such as accidents, injuries, acute psycho-emotional events, and other inflammatory processes), it is important to consider the potential confounding effects on the pain score results.

It is crucial to acknowledge the limitations of our program, which include a small sample size, short duration, and lack of long-term follow-up. However, it is worth noting that the data passed the normality test, and most of the *p*-values were <0.05, indicating statistical significance, except for the decrease in PCL-5 score. Although the study size is comparable to other early research investigating the implementation of activity-based treatments for fibromyalgia, further research is needed to elucidate potential changes in PTSD symptoms that may have been overlooked in our study.

Another limitation was missing data from incomplete questionnaires, in part due to the complexity of the MPQ, in which the completion rate was only 56% (i.e., 14 out of 25). This may have introduced selection bias, as participants who had strong feelings about their experiences were more likely to complete all questionnaires compared to those who did not. However, despite this limitation, the results remained robust based on the available data and were supported by a change in NRS pain scores with 96% completion rate (i.e., 24 out of 25) and qualitative analysis.

Furthermore, while integrative medicine models have well-established cost-effectiveness,^[26,27]^ the implementation of such approaches on a broader scale may encounter challenges due to socioeconomic disparities. Limited financial, spatial, and personnel resources can hinder the widespread adoption of this model. To address this issue, the program can be adapted for outpatient settings, where resources may be more constrained.

During our program, we encountered consistent challenges related to group appointment cancelations. To mitigate this issue in future implementations, virtual group visits, telehealth coaching, and the use of educational and instructional videos can be considered as alternatives to accommodate appointment cancelations. In addition, free mindfulness apps and resources to help with sleep and relaxation available at the VA could also reduce the socioeconomic disparities. These measures can help ensure that participants still receive the necessary support and guidance.

Moreover, for patients with severely limited physical capacity, incorporating less intensive physical activities, such as chair yoga, can be particularly beneficial. By tailoring the program to cater to individuals with restricted physical abilities, we can provide meaningful engagement and promote their well-being within their specific limitations.

To further strengthen and validate these findings, future research should consider incorporating a larger sample size, prospective data collection, and control groups. These additional measures would provide a more comprehensive understanding of the outcomes and help address potential confounding factors. By expanding the scope of investigation, we can enhance the reliability and generalizability of the results, thereby advancing our knowledge in this field.

### 4.3. Interpretation

Results of retrospective analysis showed that this integrative care model was well-received by the patients and has the potential to significantly improve QoL and pain levels. However, no statistically significant difference was found for PTSD symptoms. These results for integrative fibromyalgia treatments in the outpatient setting are consistent with the success of holistic approaches in inpatient pain management as reported in literature.^[28]^ These results demonstrate improvements in fibromyalgia related QoL, pain, and depression symptoms. They mirror the trends in results of integrative interventions in literature.^[28]^ While existing research showed that primary care settings might be ideal for the treatment of fibromyalgia,^[29]^ our results suggest a collaborative approach could improve patient empowerment, satisfaction, and outcomes.

Additional analyses showed significant positive correlation between patients’ perceptions of empowerment through multidisciplinary interventions and decreases in pain (Fig. 4A). Similarly, significant positive correlation between patient empowerment and decreased FIQ score suggested that patients who felt empowered had greater improvement in their functional status (Fig. 4B). Lastly, patient empowerment weakly correlated with improvement in depression symptoms (Fig. 4B).

Tailored integrative medicine interventions, including shared goal setting, group activities, health coaching, and education, effectively alleviated fibromyalgia-related pain, depressive symptoms, and improved QoL in women. The positive outcomes of this model positioned SVAMC as a potential design site for implementing a “Whole Health System.” Since then, significant progress has been made in implementing Whole Health for all Veterans. If future research continues to support this model as a cost-effective strategy, it could serve as a model for value-based care.^[30]^

### 4.4. Generalizability

This study offers valuable insights into the potential effectiveness of the outpatient integrative care model for fibromyalgia in women. However, the external validity of the findings may be influenced by the specific patient population, treatment setting, and sociocultural characteristics. This study included women who received care at a specific Veterans Affairs Medical Center, potentially limiting the representation of other patient groups. Moreover, since the treatment program was conducted in an outpatient setting at a specific site, the generalizability of the results to other healthcare settings, such as private clinics or community health centers, may be constrained by varying resources and patient populations. Cultural and social factors unique to the participants and the Upstate New York area where the study was conducted could also affect the generalizability to other cultural contexts. To enhance the external validity of the results, further research with larger and more diverse samples, including control groups and multi-center studies, is essential. Investigations in different healthcare settings and cultural contexts would strengthen the ability to generalize the findings and promote broader implementation of the integrative care model.

## 5. Conclusion

This paper retrospectively reports on the implementation of an integrative model for the treatment of fibromyalgia in women. This model incorporated Functional Medicine and emphasized personalized goals, patient education, non-pharmaceutical treatments, and lifestyle modifications. The results demonstrated significant levels of patient satisfaction as well as improvements in pain control, quality of life, and symptoms of depression. This integrative model is proposed as a feasible and potentially effective treatment approach for fibromyalgia in women Veterans. Future research should consider incorporating a larger sample size, prospective data collection, and control groups to validate the current results.

## Acknowledgments

We acknowledge the employment of Harminder Grewal, MD, MBBS, DGO, FAAFP, former Women Health Medical Director and Whole Health Clinical Director at SVAMC at Syracuse, NY and current Whole Health Clinical Lead at Veterans Affairs Medical Center at Dayton, OH and Chair of the Department of Family Medicine at Wright State University Boonshoft School of Medicine, Fairborn, OH.We acknowledge the employment of Catherine M. Vernon, MD, PhD, Associate Chief of Staff for Education (ACOS/E) and Designated Education Officer and Steven W. Brose, DO, Chief of Spinal Cord Injuries/Disorders at SVAMC at Syracuse, NY. We are grateful for Timothy Crawford, PhD, MPH, Associate Professor at Wright State University Boonshoft School of Medicine, Department of Family Medicine, for the contribution of his statistical expertise and the assessment of general study design and methodology. We acknowledge the contribution of Women Wellness Center SVAMC, Department of Women Health SVAMC, Department of Physical Medicine, and Rehabilitation SVAMC, Meribeth Ogrinc MD, Jana Dimitrievska - Health Coach and all medical staff that participated in this project. All acknowledged have given permission to be named.

## Author contributions

**Conceptualization:** Harminder Grewal, Bushra Atta Ur Rehman, Julia Norton, Catherine M. Vernon, Simrita Deol, Steven W. Brose.

**Data curation:** Harminder Grewal, Mahwish Iqbal, Bushra Atta Ur Rehman, Catherine M. Vernon, Steven W. Brose.

**Formal analysis:** Harminder Grewal, Mahwish Iqbal, Bushra Atta Ur Rehman, Catherine M. Vernon, Simrita Deol, Steven W. Brose.

**Funding acquisition:** Harminder Grewal.

**Investigation:** Harminder Grewal, Catherine M. Vernon, Simrita Deol.

**Methodology:** Harminder Grewal, Cindy Zhuang, Mahwish Iqbal, Bushra Atta Ur Rehman, Julia Norton, Catherine M. Vernon, Simrita Deol.

**Project administration:** Harminder Grewal, Catherine M. Vernon, Simrita Deol.

**Resources:** Harminder Grewal, Catherine M. Vernon, Steven W. Brose.

**Software:** Harminder Grewal, Steven W. Brose.

**Supervision:** Harminder Grewal, Catherine M. Vernon, Steven W. Brose.

**Validation:** Harminder Grewal, Cindy Zhuang, Mahwish Iqbal, Bushra Atta Ur Rehman, Catherine M. Vernon, Simrita Deol, Steven W. Brose.

**Visualization:** Harminder Grewal, Cindy Zhuang, Mahwish Iqbal, Bushra Atta Ur Rehman, Simrita Deol.

**Writing – original draft:** Harminder Grewal, Cindy Zhuang, Mahwish Iqbal, Bushra Atta Ur Rehman, Julia Norton, Catherine M. Vernon, Simrita Deol.

**Writing – review & editing:** Harminder Grewal, Cindy Zhuang, Simrita Deol, Steven W. Brose.
